# Supplementary material for: High-yield parallel fabrication of quantum-dot monolayer single-electron devices displaying Coulomb staircase, contacted by graphene
Source: Nat Commun. 2021 Jul 14;12:4307. doi: 10.1038/s41467-021-24233-2 (PMC8280191; doi:10.1038/s41467-021-24233-2)
Supplement: Supplementary file 1 — Supplementary Information [file 41467_2021_24233_MOESM1_ESM.pdf]

Supplementary Information for: High-yield parallel fabrication of quantum-dot monolayer single-electron devices displaying Coulomb staircase, contacted by graphene

Joel M. Fruhman[1*], Hippolyte P.A.G. Astier[1*], Bruno Ehrler[1], Marcus L. Böhm[1], Lissa F.L. Eyre[1], Piran R. Kidambi[2,3], Ugo Sassi[4], Domenico De Fazio[4], Jonathan P. Griffiths[1], Alexander J. Robson[5], Benjamin J. Robinson[5], Stephan Hofmann[2], Andrea C. Ferrari[4], Christopher J.B. Ford[1*]

[1]Cavendish Laboratory, University of Cambridge, JJ Thomson Av., Cambridge CB3 0HE, UK
[2]Department of Engineering, University of Cambridge, 9 JJ Thomson Avenue, Cambridge CB3 0FA, UK
[3]Department of Chemical and Biomolecular Engineering, Vanderbilt University, Nashville, TN 37212.
[4]Cambridge Graphene Centre, 9 JJ Thomson Avenue, Cambridge CB3 0FA, UK
[5]Department of Physics, Lancaster University, Lancaster LA1 4YB, UK
*email: joel.fruhman@cantab.net, hipp.astier@cantab.net, cjbf@cam.ac.uk

Supplementary Figures

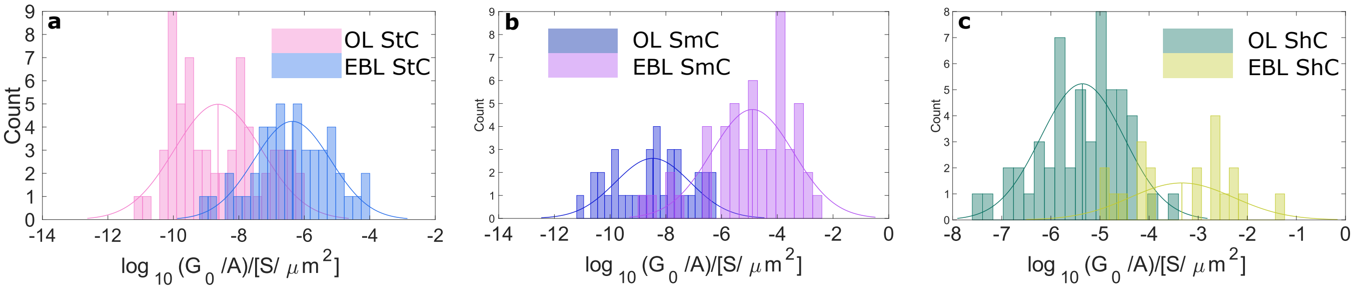

Supplementary Figure 1. Histograms of the number of devices *vs* their differential conductance at $V = 0$ (G_0) per unit area, showing an increase in G_0 for EBL relative to OL devices, for **a,** StC, **b,** SmC and **c,** ShC. The broad distribution for StC overlaps that of SmC for both OL and EBL and so some of these SmC are possibly not from conduction through C6S2, but from many QDs conducting in parallel.

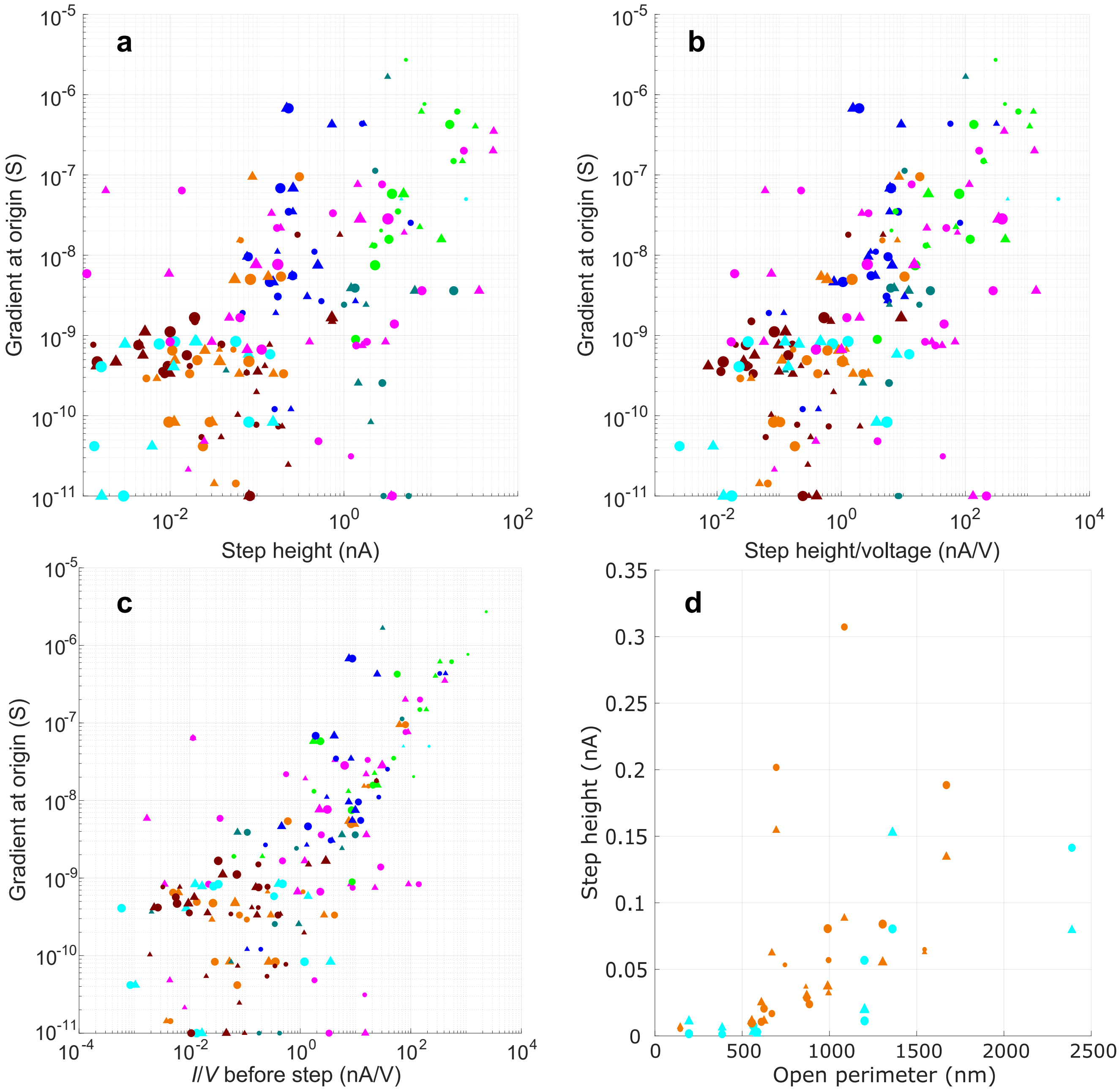

Supplementary Figure 2. Scatter plots displaying positive correlation between the differential conductance at zero bias (G_0) and **a,** current step height of first steps ΔI and **b,** the ratio of ΔI to voltage at which the step occurred ($\Delta I/V_s$). **c,** Scatter plot showing the positive correlation between G_0 and the ratio of current to voltage just before the step occurs (I_s/V_s). I_s is recorded at the base of the step riser. Steps with $G_0 < 10^{-11}$ S are shown at 10^{-11} S. **d,** Scatter plot showing ΔI *vs* open perimeter, for EBL cyan and orange trends. Correspondence between these groups, when plotted against open perimeter, suggests that in these largest EBL devices, the length of the etched edge dominates the behaviour. When the EBL areas are reduced further, device area is again the best predictor of ΔI. In all plots, symbols match those in Fig. 3a: their colours correspond to the different batches, circles (triangles) represent the steps at the smallest positive (negative) voltage, and larger data points correspond to StC of a higher quality.

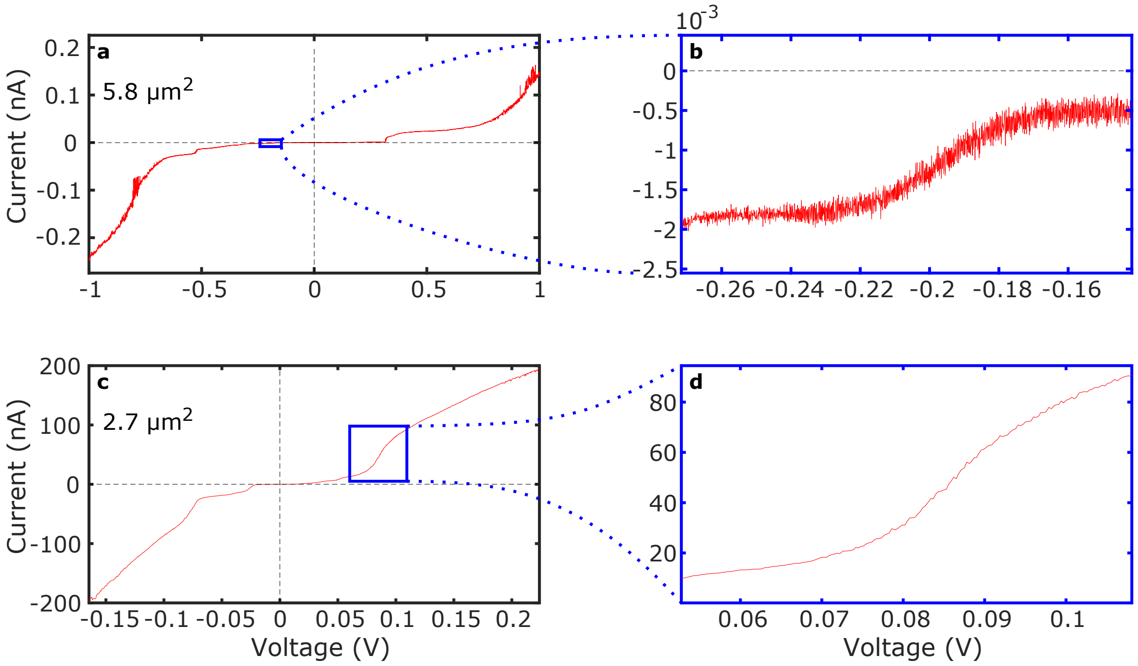

Supplementary Figure 3. **a,** A 5.8 μm^2 StC device containing the step with the smallest measured step height $\Delta I = 1.4\,$pA at $-0.19\,$V. **b,** The smallest measured ΔI in the dataset from the StC in **a**. **c,** StC from a device with a 2.7 μm^2 area containing the step with the largest measured step height $\Delta I = 85\,$nA at $0.09\,$V. **d,** The largest step height in **c**.

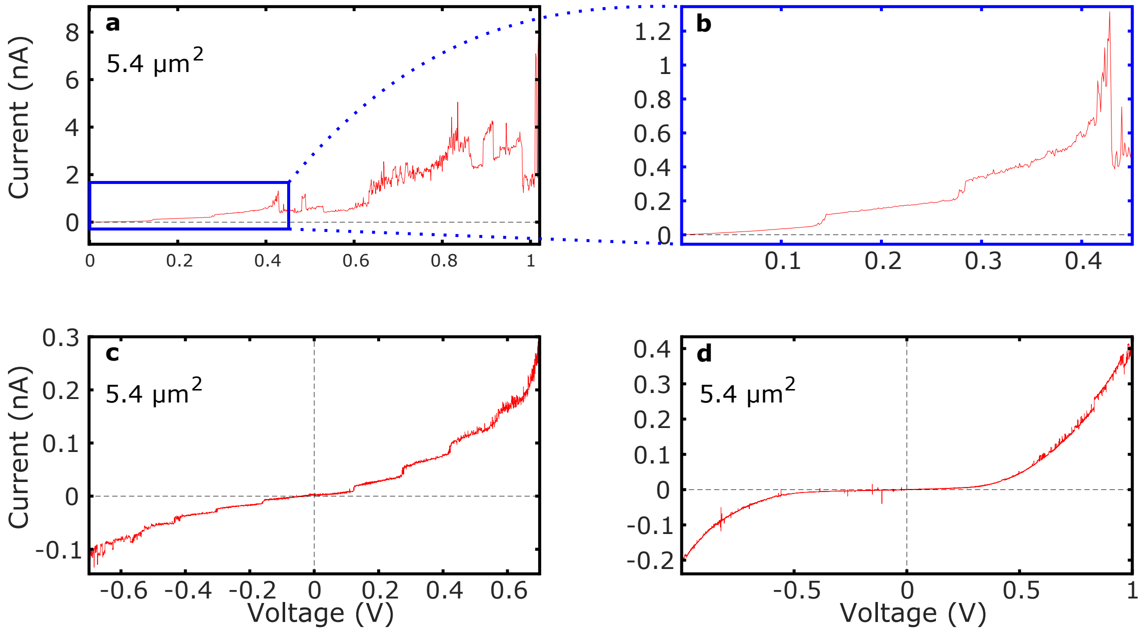

Supplementary Figure 4. StC from a 5.4 μm^2 area device that is initially unstable. The device stabilises under bias and is then thermally cycled. **a,** The first sweep beginning at $0\,$V shows successive discrete steps in current for $V < 0.4\,$V. For $V > 0.4\,$V the I–V curve displays RTN with no discernible Coulomb staircase. **b,** Inset of sweep in **a** shows two steps for $V < 0.4\,$V. **c,** A stabilised curve trace showing 12 Coulomb-staircase current steps. This is seen immediately on the return sweep that follows the measurement in **a**, in the range $-0.7\,$V to $0.7\,$V. The device behaviour for $0.4\,\text{V} < V < 0.7\,$V has stabilised and there is now clear Coulomb staircase in this region. Following these two sweeps, a further 22 I–V curves are taken that all closely resemble what is seen in **c**. **d,** After a single thermal cycle—returning the device to room temperature and then cooling it back to $4\,$K—the staircase behaviour has disappeared.

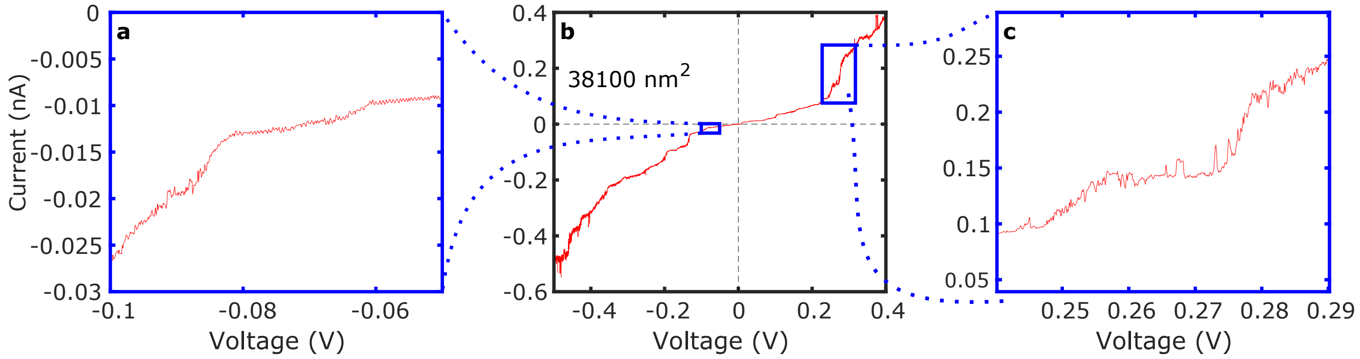

Supplementary Figure 5. EBL StC device showing a large ΔI range of 1.7–77 pA. **a,** The smallest step in the trace, at -0.06 V. **b,** A full voltage sweep, representative of the 50 taken, showing a cascade of 35 steps of varying heights and widths in the range $-0.5\,\text{V} < V < 0.4\,\text{V}$ **c,** The largest step at 0.3 V.

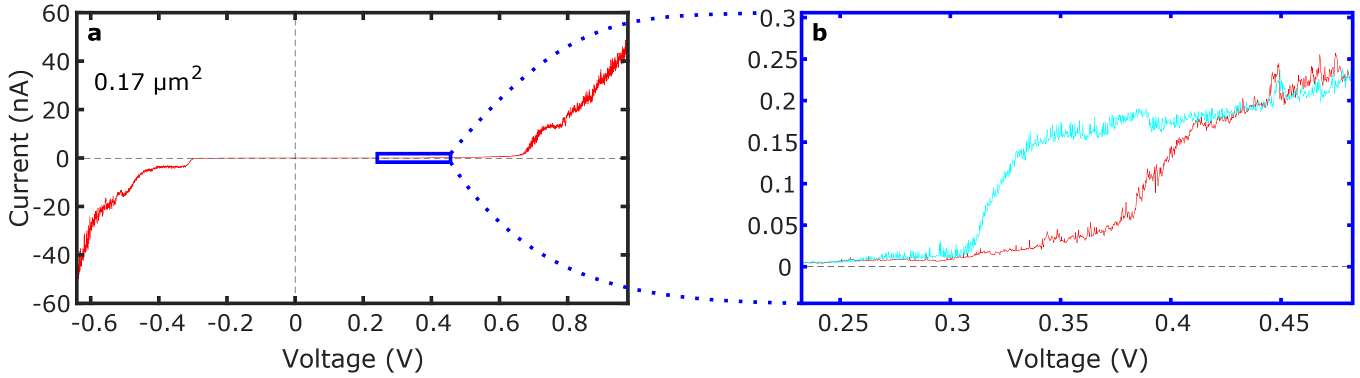

Supplementary Figure 6. EBL device showing a permanent lateral shift in the curve trace that remains over many sweeps. **a,** Example overall trace. **b,** The shift of 0.065 V, which occurs only around the step at 0.36 V and is not seen throughout the rest of the I–V curve. Both sweeps are in the same direction, from positive to negative voltage.

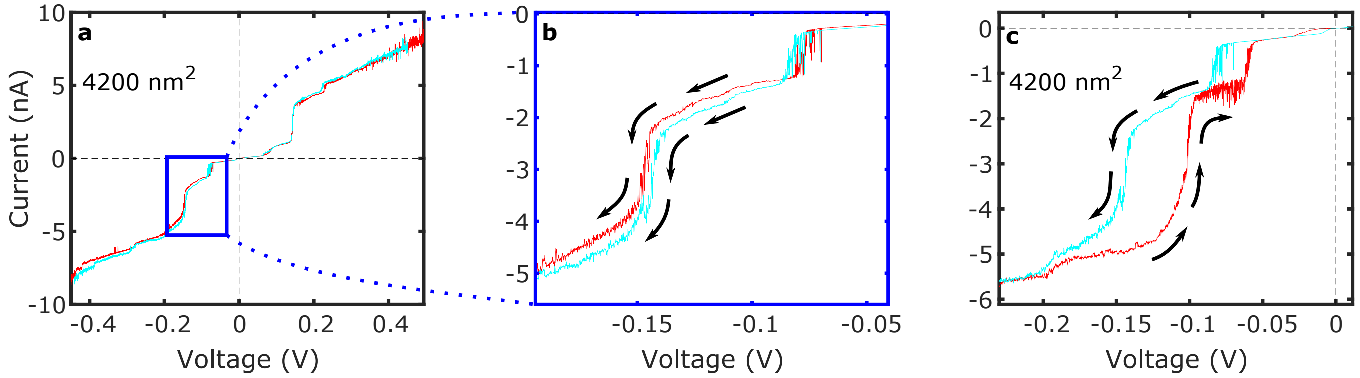

Supplementary Figure 7. **a,** EBL device with 4200 nm^2 area displaying 16 steps with a high G_0 of $7.6 \times 10^{-9}\,\text{S}/\mu\text{m}^2$. **b,** Lateral shifts in opposite directions for two adjacent steps traced with the same sweep direction. RTN can be seen on the step at -0.08 V. **c,** The same two steps maintain a sweep-direction-dependent lateral shift of ~ 0.04 V. Sweep directions are shown with arrows by each curve.

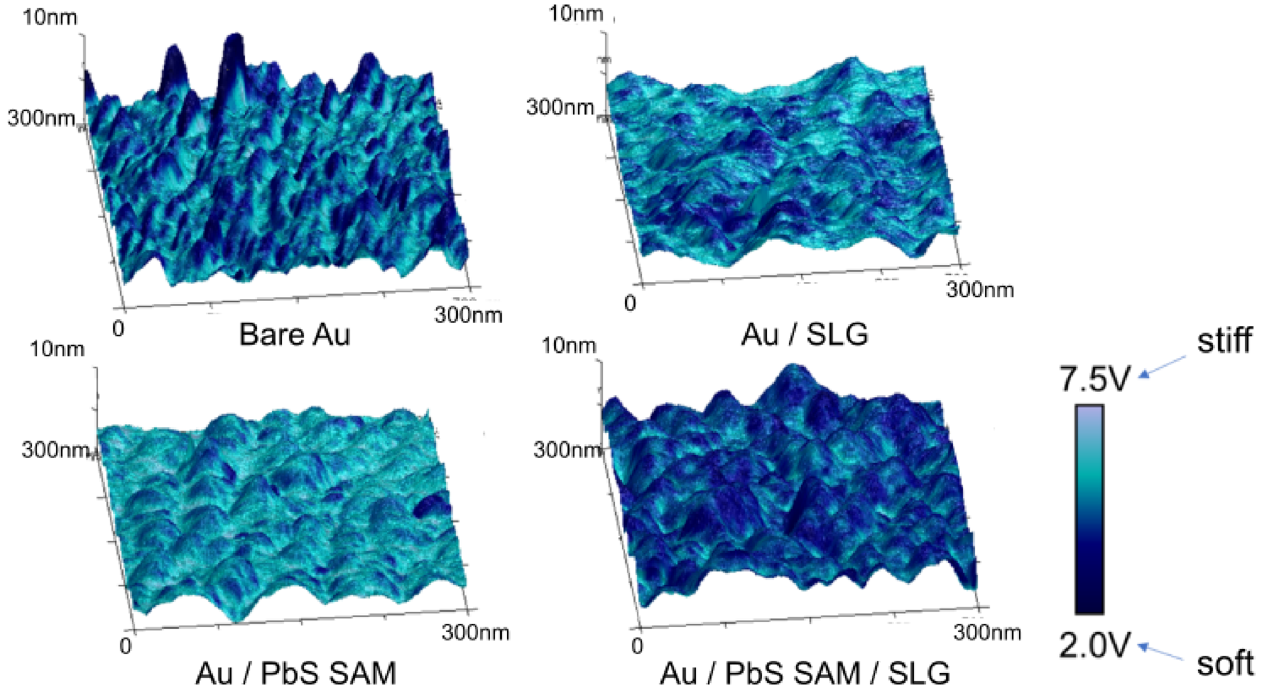

Supplementary Figure 8. Combined AFM with UFM in four different fabrication states. Bare Au, Au with SLG transferred directly on top, Au with the QD SAM assembled on top and Au with the QD SAM assembled and SLG transferred. The device topographies are represented in the regular three-dimensional spatial axes and the UFM data, showing mechanical stiffness, is displayed in the overlaid colour-map. Moving from bare Au to Au/SLG and Au/PbS-SAM shows an increase in mechanical stiffness, but when SLG is transferred on top of the SAM, the stiffness decreases significantly

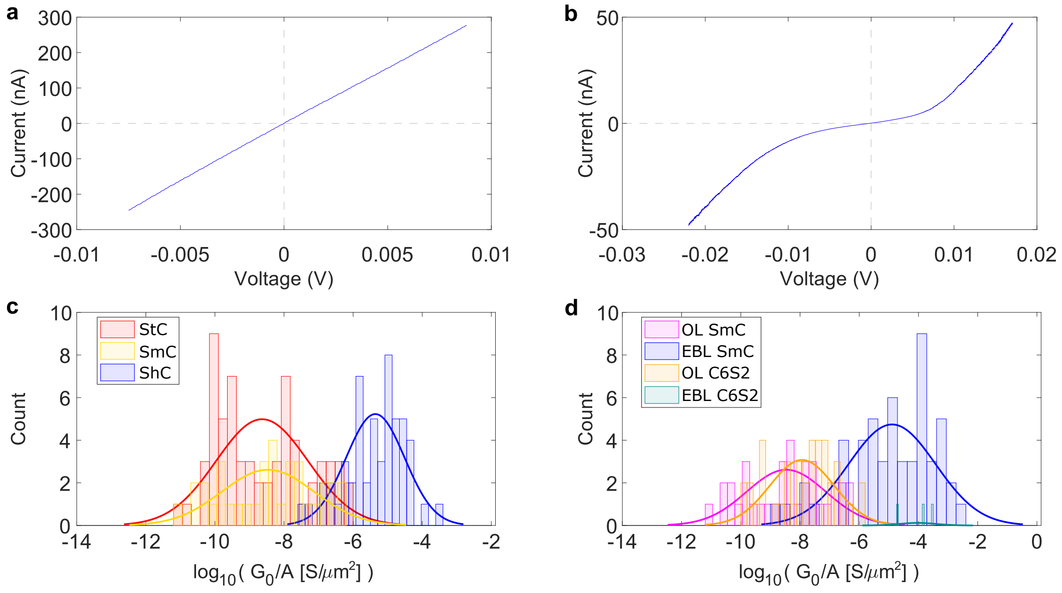

Supplementary Figure 9. **a,** Typical short-circuit curve (ShC) with $\sim 30\,\mathrm{k\Omega}$ resistance. **b,** A smooth curve (SmC) that does not display current plateaux but is clearly non-Ohmic. **c,** Histograms of the low-voltage conductance per unit area of OL curve types. **d,** Histograms of the low-voltage conductance per unit area of OL SmC and EBL SmC together with the OL C6S2 and EBL C6S2 measured from the control devices in separate overlapping distributions (ShC from the C6S2 devices removed).

Supplementary Table 1: Comparison of literature results on devices and conductive-probe experiments producing Coulomb blockade

Junction structure	Device or conductive-probe experiment D/CP	Requires immobilisation of single nanoparticles Y/N	Requires EBL-defined QD object Y/N	Requires prior fabrication of a nanogap Y/N	Gate-defined Coulomb island Y/N	Scalable processes[1] Y/N	Yield of Coulomb-blockade devices	Reference
Au - C6S2 - PbS QD - OA ligand - SLG	D	N	N	N	N	Y	$87 \pm 13\%$[2]	This work
Ag/Pd - CNT[3] - Ag QD - CN - Ag/Pd	D	Y	N	N	N	N	NC[4]	[1]
Au - C*n*S2 - Au NP - C*n*S2 - Au	D	Y	N	Y	N	N	NC. Nanogap yield[5] = 41%[2], 90%[3, 4]	[2–6]
Au - 3-leg-phenol - Sn-por[6] protected Au NP - 3-leg-phenol - Au	D	Y	N	Y	N	N	NC	[7]
Graphene – Graphene QD – Graphene	D	Y	N	Y	N	N	NC	[8]
Ti/Au - MoS_2 nanoribbon – Ti/Au	D	Y	Y	N	N	N	NC	[9]
Ti/Au - Bi_2Te_3 – Ti/Au	D	N	Y	N	N	N	NC	[10]
Gate-defined bilayer graphene	D	N	Y	N	Y	N	NC	[11]
Graphene – aromatic-ring molecule - graphene	D	Y	N	Y	N	N	40%	[12]
Metallic contacts - Metallic island/ AlGaAs 2DEG[7] – Metallic contacts	D	N	Y	N	Y	N	NC	[13]
Au - Au NPs - Au	D	Y	Y	Y	N	N	15-20%	[14]
Pt - single molecule - Pt	D	Y	N	Y	N	N	NC	[15, 16]
Si - Si QD - Si	D	N	Y	N	N	N	NC	[17]
Si - Si QD - Si	D	N	Y	N	N	N	50%	[18]
Ti/Pt - CNT - Ti/Pt	D	Y	N	N	N	N	NC	[19]
Au - C*n*S2 - Au NP - C*n*S2 - Au	D	Y	Y	Y	N	N	∼10%	[20]
Cr-Au NP - Cr	D	Y	N	Y	N	N	∼ 1%	[21]
Au/Ti - Apoferritin-caged Co_3O_4/InO NP - Au/Ti	D	Y	N	Y	N	N	NC	[22]
Au - CF_2 - Au NP - CF_2 - Au	D	Y	N	Y	N	N	NC	[23]
Pt - SiO_2 - Au NP - SiO_2	D	N	N	Y	N	N	NC	[24]
Ti/Au - CNT in Au NP:Al_2O_3 - Ti/Au	D	Y	N	Y (∼300 nm, length of CNT)	N	N	NC	[25]
Cr/Au - pentacene - Au NP - pentacene	D	Y	N	Y	N	N	37%	[26]

[1] Is compatible with current mass-manufacturing methods
[2] For device areas $\leq 2\,\mu m^2$
[3] CNT: carbon nanotube
[4] NC: not communicated in the publication, to the best of our knowledge
[5] No device yield. i.e. The fabrication yield of the nanogap (given) multiplied by the QD immobilisation yield in working nanogaps (not given)
[6] Sn-por: Sn-porphyrin
[7] 2DEG: 2-dimensional electron gas

Junction structure	Device or conductive-probe experiment D/CP	Requires immobilisation of single nanoparticles Y/N	Requires EBL-defined QD object Y/N	Requires prior fabrication of a nanogap Y/N	Gate-defined Coulomb island Y/N	Scalable processes Y/N	Yield of Coulomb blockade devices	Reference
Pt STM[8] - hexyl ligands - Si NP - hexyl ligands - Au	CP	NA[9]	NA	NA	NA	NA	NA	[27]
W STM tip - $Co_{30}Fe_{70}$ NP - MgO - $Co_{40}Fe_{40}B_{20}$	CP	NA	NA	NA	NA	NA	NA	[28]
CP - vacuum - C*n*S - Au QD - C*n*S - Au	CP	NA	NA	NA	NA	NA	NA	[29]
CP - vacuum - TBA[10] ligand - Au NP - TBA ligand - thiolactic acid - Au	CP	NA	NA	NA	NA	NA	NA	[30]
CP - vacuum - OPE[11] - Au NP - OPE - Au	CP	NA	NA	NA	NA	NA	NA	[31]
STM - Au NP - molecular SAM - Si	CP	NA	NA	NA	NA	NA	NA	[32]
STM - Sn NP - STO[12]	CP	NA	NA	NA	NA	NA	NA	[33]

[8] STM: scanning tunnelling microscope
[9] NA: not applicable (e.g. not relevant to the structure or CP-type experiments)
[10] TBA: bis-4,4'-(4,4'-dithiobutylbenzyl)-N,N,N',N'-tetraethylamine
[11] OPE: Oligo(phenylene-ethynylene)
[12] STO: silicon tin oxide

Supplementary Notes

Supplementary Note 1. OL and EBL Comparison

Across their entire area ranges, OL and EBL devices produce the same StC yield $\sim$ 42%, but EBL devices have fewer shorts, with a successful junction yield (StC or SmC) $\sim$84%, compared to $\sim$65% in OL devices. The similar StC yields between lithography types is surprising because an extrapolation of the StC success, from the OL regression analysis, would suggest a further increase in StC yield for EBL devices due to their smaller area (confirmed with SEM imaging, Fig. 1h). In addition, an overall increase in conduction per unit area is seen with the EBL devices (Supplementary Fig. 1). All these results taken together may be because the EBL devices have larger open (etched) SLG edge-to-area ratios than OL devices. The EBL (Fig. 1c) devices have three open edges and one closed (where the SLG overlaps the Au electrode edge and goes down its vertical side), a ratio that is reversed for OL. Having a greater proportion of the top SLG electrode edge free and unpinned from the Au may reduce the SLG's tenting effect and result in more contact with the underlying NPs. This would explain the higher conduction per unit area in EBL devices. Furthermore, having proportionately less closed edge may lower the probability of shorts, explaining the increase in junction yield. The fact that the reduced number of shorts translates not into a proportionately higher StC yield, but into an increased percentage of SmC, could also be a consequence of the top electrode being in better electrical contact. Here, the dominant Coulomb-blockade behaviour, discussed in the main text, is more likely to be washed out due to increased parallel conduction through multiple different-sized QDs, which have different step voltages.

Supplementary Note 2. StC group variation and device stability

Devices are selected for the StC group when they display a recognisable Coulomb-staircase curve type but there is significant variation of electronic behaviour within the group. Step heights (ΔI) across the StC group span at least four orders of magnitude (Fig. 3) and can be seen within a single device spanning more than one order of magnitude; 1.7–77 pA in Supplementary Fig. 5. Similarly, the voltage increase required to induce successive current steps (ΔV) is not constant between or within devices (Fig. 2). Differential conductance at 0 V (G_0) per unit area of these devices ranges from 6.4 pS/μm^2 to 83 μS/μm^2 (Supplementary Fig. 1a). A very high level of electronic stability is seen in almost all Coulomb-staircase devices even after a thermal cycle. Some are swept hundreds of times and remain unaltered, save for minor lateral shifts in their traces. Some shifts that occur after a certain number of sweeps or after a specific magnitude of source-drain bias is reached, are permanent and independent of sweep direction (Supplementary Fig. 6). Other curves show both positive and negative shifts at different steps along the trace (Supplementary Fig. 7). There are also lateral step shifts that are dependent on sweep direction (Supplementary Fig. 7). All these changes are likely to result from charge trapping, which acts like a gate voltage.

Low-frequency RTN is seen across most StC: this likely results from either mechanical instability that causes the junctions to oscillate between conduction configurations under bias[34], or surface states trapping charges over long timescales. The large forces endured by initially unstable devices, i.e. devices with a lot of RTN, sometimes causes permanent changes to the junction's conduction and staircase structure. Often these changes lead to increased stability, suggesting that the forces settle unstable device components, such as free unbonded QDs or loose SLG flaps (Supplementary Fig. 4).

Supplementary Note 3. Confirmation of graphene suspension

SLG suspension is confirmed by comparing stiffness measurements of devices with and without the SLG top electrode, using ultrasonic force microscopy (UFM)[35]. We observe a marked reduction in the mechanical stiffness in SLG-covered NP SAM devices (Supplementary Fig. 8). We expect that, given the large forces exerted when a bias is applied across a device and the ease with which the oleic-acid capping ligand can bend[36], the effective

resistance of the dominant double-barrier structures may decrease during measurements, reinforcing the preferred current pathway. This is corroborated by the fact that, in general, the step height ΔI increases with $|V|$ within individual devices (Supplementary Fig. 5). This relation is maintained over many sweeps, so any physical change to the top barrier at high $|V|$ is reversible. This points to an elasticity that accompanies the top electrode's flexibility, supporting the idea that these properties are likely a key mechanism for minimising the number of active QDs.

The formation of peaks and troughs in the Au topography, which are a result of vacuum evaporation, in combination with a flexible top barrier, could go some way to explaining how electronic behaviour associated with low numbers of QDs occurs frequently in large-area devices.

Supplementary Note 4. Step fitting

Hyperbolic tangent functions are fitted to all individual steps across all StC to gather step characteristics in a database using:

$$I = I_0 + \frac{I_\mathrm{h}}{2} \tanh \frac{V - V_\mathrm{s}}{\Delta V} + \Theta(V - V_\mathrm{s}) g_0 (V - V_\mathrm{s}) + \Theta(V_\mathrm{s} - V) g_1 (V - V_\mathrm{s}), \quad (1)$$

where I_0 is the current at centre of the riser, I_h is the amplitude of the riser, V_s is the voltage at the centre of the riser, g_0 (g_1) is the current gradient at voltages less (greater) than V_s, and $\Theta(x)$ is the Heaviside function.

The data and fits show steps with a finite gradient and rounded edges. These occur for finite unequal tunnelling rates into and out of the QD[37]. These features are unlikely to be the result of many QDs contributing to a single current step because the voltages at which the steps arise vary significantly between devices.

Supplementary Note 5. Batch dependence and clustering in Step Height–Area correlations

In order to see the correlation between step height and device area, the data must be separated into fabrication batches. Whilst the same QDs are used in all batches, this grouping implies that variations in batch processing, such as surface contamination, minor changes in the lithography and assembly of each batch, have a global effect on the conduction of junctions in a given batch.

These batch-dependent trends are themselves grouped into discrete step-height clusters (Fig. 3a-d). In Fig. 3a, OL batch 1 is offset laterally by a factor 100 for clarity. Step heights of junctions of a given area are generally a factor of about two higher than for OL batch 2, except for an additional lower-conduction group seen only in batch 2. The EBL data show the smallest steps, the lower bound being set by the equipment resolution. In general, the EBL set contains StC of a higher quality than those produced with OL, but the cyan cluster is the best trend observed. Here, all the underlying StC show many clear, sharp steps, without random telegraph noise. These areas are designed to sit between OL and EBL area sizes and provide dataset continuity, but they form a trend in their own right.

When the step heights in the EBL data are plotted against open (etched) perimeter, the cyan lines up with the orange trend suggesting that there may be some threshold of the etched SLG edge-to-area ratio above which the length of the open edge determines a device's step heights, rather than the area (Supplementary Fig. 2d).

Supplementary References

[1] Zhang, J. *et al.* Room-Temperature Carbon Nanotube Single-Electron Transistors with Mechanical Buckling–Defined Quantum Dots. *Adv. Electron. Mater.* **4**, 1700628 (2018).

[2] Azuma, Y. *et al.* Single-Electron Transistor Fabricated by Two Bottom-Up Processes of Electroless Au Plating and Chemisorption of Au Nanoparticle. *Jpn. J. Appl. Phys.* **49**, 90206 (2010).

[3] Serdio V., V. M. *et al.* Robust nanogap electrodes by self-terminating electroless gold plating. *Nanoscale* **4**, 7161–7167 (2012).

[4] Serdio V, V. M. *et al.* Gap separation-controlled nanogap electrodes by molecular ruler electroless gold plating. *RSC Adv.* **5**, 22160–22167 (2015).

[5] Azuma, Y., Onuma, Y., Sakamoto, M., Teranishi, T. & Majima, Y. Rhombic Coulomb diamonds in a single-electron transistor based on an Au nanoparticle chemically anchored at both ends. *Nanoscale* **8**, 4720–4726 (2016).

[6] Kano, S. *et al.* Ideal Discrete Energy Levels in Synthesized Au Nanoparticles for Chemically Assembled Single-Electron Transistors. *ACS Nano* **6**, 9972–9977 (2012).

[7] Vivitasari, P. U., Azuma, Y., Sakamoto, M., Teranishi, T. & Majima, Y. Coulomb blockade and Coulomb staircase behavior observed at room temperature. *Mater. Res. Express* **4**, 24004 (2017).

[8] Fried, J. P. *et al.* Large amplitude charge noise and random telegraph fluctuations in room-temperature graphene single-electron transistors. *Nanoscale* **12**, 871–876 (2020).

[9] Kotekar-Patil, D., Deng, J., Wong, S. L. & Goh, K. E. J. Coulomb Blockade in Etched Single- and Few-Layer MoS2 Nanoribbons. *ACS Appl. Electron. Mater.* **1**, 2202–2207 (2019).

[10] Jing, Y. *et al.* A Single-Electron Transistor Made of a 3D Topological Insulator Nanoplate. *Adv. Mater.* **31**, 1903686 (2019).

[11] Kurzmann, A. *et al.* Charge Detection in Gate-Defined Bilayer Graphene Quantum Dots. *Nano Lett.* **19**, 5216–5221 (2019).

[12] Xu, Q. *et al.* Single Electron Transistor with Single Aromatic Ring Molecule Covalently Connected to Graphene Nanogaps. *Nano Lett.* **17**, 5335–5341 (2017).

[13] Jezouin, S. *et al.* Controlling charge quantization with quantum fluctuations. *Nat.* **536**, 58–62 (2016).

[14] Bolotin, K. I., Kuemmeth, F., Pasupathy, A. N. & Ralph, D. C. Metal-nanoparticle single-electron transistors fabricated using electromigration. *Appl. Phys. Lett.* **84**, 3154–3156 (2004).

[15] Choi, Y. Y., Kwon, A. & Majima, Y. Heteroepitaxial spherical electroless Au-plated Pt-based nanogap electrodes of radius 5 nm and gap separation 0.7 nm. *Appl. Phys. Express* **12**, 125003 (2019).

[16] Lee, S. J. *et al.* Single-molecule single-electron transistor (SM-SET) based on π-conjugated quinoidal-fused oligosilole and heteroepitaxial spherical Au/Pt nanogap electrodes. *Appl. Phys. Express* **12**, 125007 (2019).

[17] Shin, S. J. *et al.* Si-based ultrasmall multi-switching single-electron transistor operating at room-temperature. *Appl. Phys. Lett.* **97**, 103101 (2010).

[18] Lee, S., Lee, Y., Song, E. B. & Hiramoto, T. Observation of Single Electron Transport via Multiple Quantum States of a Silicon Quantum Dot at Room Temperature. *Nano Lett.* **14**, 71–77 (2014).

[19] Maeda, M., Iwasaki, S., Kamimura, T., Murata, K. & Matsumoto, K. Room-Temperature Carbon Nanotube

Single-Electron Transistor with Defects Introduced by La({NO}3)3Process. *Jpn. J. Appl. Phys.* **47**, 5724–5726 (2008).

[20] Luo, K., Chae, D.-H. & Yao, Z. Room-temperature single-electron transistors using alkanedithiols. *Nanotechnol.* **18**, 465203 (2007).

[21] Ray, V. *et al.* CMOS-compatible fabrication of room-temperature single-electron devices. *Nat. Nanotechnol.* **3**, 603–608 (2008).

[22] Kumagai, S. *et al.* Self-aligned placement of biologically synthesized Coulomb islands within nanogap electrodes for single electron transistor. *Appl. Phys. Lett.* **94**, 83103 (2009).

[23] Kim, C., Kim, H. S., Qin, H. & Blick, R. H. Coulomb-Controlled Single Electron Field Emission via a Freely Suspended Metallic Island. *Nano Lett.* **10**, 615–619 (2010).

[24] Zhang, X. A. *et al.* Single electron transistor with programmable tunnelling structure. *Phys. Lett. A* **374**, 4880–4884 (2010).

[25] Seike, K. *et al.* Carbon nanotube single-electron transistors with single-electron charge storages. *Jpn. J. Appl. Phys.* **54**, 06FF05 (2015).

[26] Zheng, H. *et al.* Room temperature Coulomb blockade effects in Au nanocluster/pentacene single electron transistors. *Nanotechnol.* **26**, 355204 (2015).

[27] Basu, T. S., Diesch, S. & Scheer, E. Single-electron transport through stabilised silicon nanocrystals. *Nanoscale* **10**, 13949–13958 (2018).

[28] Temple, R. C., McLaren, M., Brydson, R. M. D., Hickey, B. J. & Marrows, C. H. Long spin lifetime and large barrier polarisation in single electron transport through a CoFe nanoparticle. *Sci. Reports* **6**, 28296 (2016).

[29] Zhang, H., Yasutake, Y., Shichibu, Y., Teranishi, T. & Majima, Y. Tunneling resistance of double-barrier tunneling structures with an alkanethiol-protected Au nanoparticle. *Phys. Rev. B* **72**, 205441 (2005).

[30] Kano, S., Azuma, Y., Kanehara, M., Teranishi, T. & Majima, Y. Room-Temperature Coulomb Blockade from Chemically Synthesized Au Nanoparticles Stabilized by Acid{\textendash}Base Interaction. *Appl. Phys. Express* **3**, 105003 (2010).

[31] Koo, H. *et al.* Characterization of thiol-functionalized oligo(phenylene-ethynylene)-protected Au nanoparticles by scanning tunneling microscopy and spectroscopy. *Appl. Phys. Lett.* **101**, 83115 (2012).

[32] Caillard, L. *et al.* Gold Nanoparticles on Oxide-Free Silicon–Molecule Interface for Single Electron Transport. *Langmuir* **29**, 5066–5073 (2013).

[33] Qin, J. *et al.* Coupling of superconductivity and Coulomb blockade in Sn nanoparticles. *Nanotechnol.* **31**, 305708 (2020).

[34] Gorelik, L. Y. *et al.* Shuttle Mechanism for Charge Transfer in Coulomb Blockade Nanostructures. *Phys. Rev. Lett.* **80**, 4526–4529 (1998).

[35] Robinson, B. J. & Kolosov, O. V. Probing nanoscale graphene–liquid interfacial interactions via ultrasonic force spectroscopy. *Nanoscale* **6**, 10806–10816 (2014).

[36] Gunawan, A. A. *et al.* Ligands in PbSe Nanocrystals: Characterizations and Plasmonic Interactions. *Microsc. Microanal.* **19**, 1506–1507 (2013).

[37] Hanna, A. E. & Tinkham, M. Variation of the Coulomb staircase in a two-junction system by fractional electron charge. *Phys. Rev. B* **44**, 5919–5922 (1991).
